# Graphene Oxide-Sensitized Surface Plasmon Resonance Biosensor of Porcine Reproductive and Respiratory Syndrome Virus

**DOI:** 10.3390/molecules27123942

**Published:** 2022-06-20

**Authors:** Xuemei Liu, Chao Xu, Chunyu Fu, Dongfang Xia, Fuchao Wang, Hongzong Yin, Jun Peng

**Affiliations:** 1 Shandong Provincial Key Laboratory of Animal Biotechnology and Disease Control and Prevention, College of Veterinary Medicine, Shandong Agricultural University, Taian 271018, China; qian1214876244@163.com (X.L.); lhr980222@163.com (C.F.); 2 College of Chemistry and Material Science, Shandong Agricultural University, Taian 271018, China; xuc@sdau.edu.cn (C.X.); xiadf@ihep.ac.cn (D.X.); 15610303603@163.com (F.W.)

**Keywords:** GO, PRRSV, FT-SPR, sensitization

## Abstract

Biosensor analysis based on the surface plasmon resonance (SPR) phenomenon enables label-free, highly sensitive analyte detection without prior sample purification or processing. However, potential applications of SPR biosensors in virus detection in biological samples remain to be explored. Owing to its excellent biocompatibility and abundance of hydroxyl and carboxyl functional groups, graphene oxide (GO) has been widely used as a biosensor of proteins and metal ions in living cells. The present work explored the effect of GO modification on the sensitivity of an SPR biosensor and used a GO-modified sensor to detect porcine reproductive and respiratory syndrome virus in cell culture, as shown. The GO modification markedly enhanced the sensitivity of the Fourier transform SPR sensor and enabled linear detection of porcine reproductive and respiratory syndrome virus (PRRSV) with a multiplicity of infection in the range 0.2–1.7 (*R*^2^ = 0.998). Such a GO-modified sensor provides a promising alternative for virus detection.

## 1. Introduction

Surface plasmon resonance (SPR) detects molecular adsorption events by measuring changes in the resonance of surface plasmon waves [1]. SPR biosensors have been developed to distinguish different target analytes and have become a conventional analytical tool for real-time, label-free analysis of biomolecular interactions [2]. Detection of protein adsorption depends on a change of the SPR signal in response to real-time interactions with materials on the sensor chip [3]. In the past two decades, SPR has been applied to many areas, such as medical diagnostics [4], food safety [5], biotechnology [6], and protein–protein interactions [5]. However, its low sensitivity to small molecules seriously hinders its application to these targets. Therefore, many new methods have been developed to enhance the SPR signal [7], and notable progress has recently been made using nanomaterials.

The large surface area and unique chemical properties of graphene oxide (GO) can be exploited to develop sensitive SPR biosensor interfaces with a strong capacity for target immobilization [8,9]. GO has been used for the analysis and detection of numerous targets, such as proteins, viable cells, and glucose [10]. SPR biosensor chips have been used to study a wide range of antibody–antigen interactions and improve the sensitivity of biosensors using GO linking layers. GO is most commonly coated on the biosensor surface using spray or immersion methods [11]. The spray method yields a higher sensitivity than the immersion method but is a time consuming and complex process. Thus, we sought to identify conditions that improve the sensitivity of SPR biosensors using simple methods and non-specialist instrumentation.

Porcine reproductive and respiratory syndrome virus (PRRSV) is a forward single-stranded RNA virus [12], which belongs to the family *Arteriviridae* [13]. PRRSV has caused huge economic losses in the global pig industry [14], and its high mortality, strong transmission, multiple transmission routes, and strong mutagenicity [15] have had a significant impact on the pig industry in China. Traditional PRRSV detection techniques include quantitative reverse transcription polymerase chain reaction (qRT-PCR), enzyme-linked immunosorbent assay (ELISA), immunohistochemistry, antibody detection, and so on [16,17,18]. These methods are time-consuming and virus sample damage treatment. Hence, developing a real-time dynamic monitoring and detection method and realizing nondestructive tests of viruses is of great significance for studying the interaction between viruses and biomolecules or antivirus drugs.

**Scheme 1:**
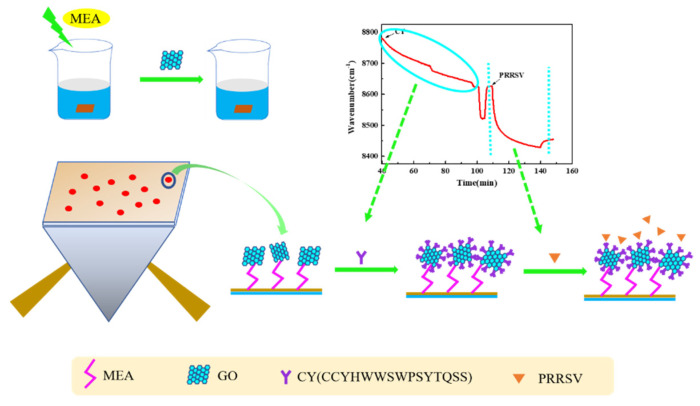
Procedure for the preparation and application of GO-based SPR biosensors.

In the present work, as shown in Scheme 1, a GO−based SPR biosensor was constructed. The sensitivity enhancement of SPR biosensors was explored using GO. After identifying GO modifications resulting in optimum sensitization, the sensor was used to detect PRRSV in mock samples.

## 2. Material and Methods

### 2.1. Construction of a GO-Based SPR Biosensor

The GO-based SPR biosensor was constructed as shown in Scheme 1. Briefly, the naked gold SPR chip was obtained from Thermo Fisher Scientific, Waltham, MA, USA. The obtained SPR chip (Thermo Fisher Scientific) was immersed in a freshly prepared piranha solution (30% H_2_O_2_:H_2_SO_4_, 3:7 *v*/*v*) for 10 min. During this period, the chip was shaken periodically to remove surface air bubbles. The chip was then rinsed thoroughly with ultrapure water, dried under N_2_ flow, then immersed in a 100 mM cysteamine hydrochloride solution (Macklin Biotech Ltd., Shanghai, China) with parafilm and stored in the dark for 12 h. Then the cysteamine hydrochloride was modified on the naked gold SPR chip by the strong interaction between sulfhydryl and gold, and the amino functional group of cysteamine hydrochloride was exposed to the surface of the chip. Finally, it was soaked in GO (XFNANO, Nanjing, China) to form a gold–cysteamine GO sensor.

In order to investigate the influence of modification conditions on SPR biosensor properties, the GO concentration, mode, and time of addition were varied to detect papain, respectively. After identifying the optimum conditions, the GO-modified sensor was probed using SEM, and the functional groups on the GO were characterized using FTIR spectroscopy.

### 2.2. Investigation of Sensitization Effect

As a mercapto protease enzyme, its papain sulfhydryl shows a strong affinity to the surface of the bare gold. In order to investigate the sensitization effect of GO and explore the possible mechanism of this sensitization, the papain was employed to investigate the sensitization effect of GO. The experimental process is briefly described as follows: Firstly, using the optimal conditions for fabrication, the GO-modified sensor was mounted in the SPR instrument and equilibrated with phosphate-buffered saline for 15 min. After the bioconjunction regents, 1-ethyl-3-(3-dimethylaminopropyl) carbodiimide (EDC) and Nhydroxysuccinimide hydrochloride (NHS) were employed to activate the surface carboxyl group of GO, and the activated GO were modified on the SPR chip by the interaction between the activated carboxyl group and the amino functional group of CY polypeptide (Sequence: NH_2_−CCYHWWSWPSYTQSS−COOH, Wuhan Xinghao Pharmaceutical Co., Ltd., Wuhan, China, abbreviated as CY). To stabilize the signal, the unreacted carboxyl groups in GO were banned using ethanolamine hydrochloride. The SPR signal was then measured in response to papain. To determine the GO-induced signal enhancement, the procedure was repeated using without GO modification.

### 2.3. Evaluates MOI Values of PRRSV

MARC-145 cells were inoculated into 96−well plates and cultured to 80%. Serial tenfold dilutions of PRRSV solution were made with DMEM. The diluted virus was inoculated into 96-well plates. Inoculate one longitudinal row per dilution, 100 µL per well. Normal cells were used as control. The results were observed and recorded day by day for 5 to 7 days. Results were calculated according to the Reed–Muench method. MOI values were calculated as PUFs = 0.7 × TCID_50_, MOI = (PUF/mL) × V_PRRSV_/N_MACK-145_, where V_PRRSV_ is the Volume of PRRSV, and N_MACK-145_ is the number of cells.

### 2.4. Proliferation and Detection of PRRSV

African green monkey kidney cells (MARC-145) were cultured in Dulbecco’s modified Eagle medium containing 10% fetal bovine serum and infected with PRRSV (JXA1 strain; GenBank accession No. EF112445.1). When the cell lesion rate exceeded 80%, the cells were lysed using three freeze–thaw cycles, cell debris was removed by centrifugation, and the supernatant was collected and stored at −80 °C until further use. 

The amount of virus required to cause lesions in 50% of the cells was calculated by the TCID_50_ (half of tissue culture infection dose) assay. This was used to calculate the viral titer (MOI).

The GO-modified sensor was mounted in the SPR instrument, and solutions containing the carbodiimide/*N*-hydroxysuccinimide activator, CY polypeptide (Sequence: NH_2_−CCYHWWSWPSYTQSS−COOH, Wuhan Xinghao Pharmaceutical Co., Ltd., abbreviated as CY), ethanolamine quencher, and PRRSV (in medium containing 10% serum and 1% double antibiotics) were added in turn. As a control experiment, the procedure was repeated using a PRRSV-free medium. 

To determine the GO-induced signal enhancement, the measurements were repeated using an SPR sensor without GO modification. 

### 2.5. Specificity of PRRSV Detection

To demonstrate that PRRSV specifically binds to CY peptides, the SPR signal in response to porcine circovirus was also measured under the same conditions. Briefly, GO-modified sensors prepared under optimal conditions were utilized. After the CY polypeptides were modified on the GO-modified SPR chip with the bioconjunction regents, EDC and NHS, and the unreacted carboxyl groups in GO were banned using ethanolamine hydrochloride. The SPR signal was then measured in response to PCV.

## 3. Results and Discussion

### 3.1. Characterization of GO

**Figure 1:**
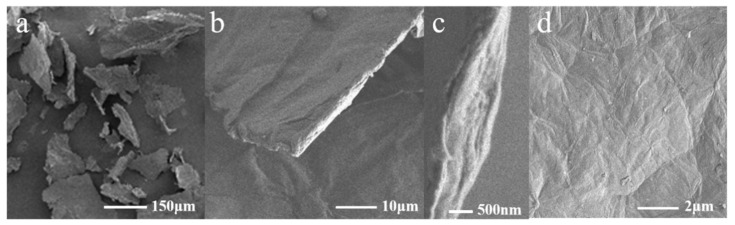
SEM images of the graphene oxide (GO) used to sensitize the surface plasmon resonance (SPR) biosensor. (**a**) Overall morphology of GO; (**b**,**c**) GO showing sheet-like structure; (**d**) GO surface with folds.

Figure 1a shows the morphology of the GO precursor material, which had a sheet structure (Figure 1b,c) and a wrinkled surface (Figure 1d), consistent with prior reports [19].

**Figure 2:**
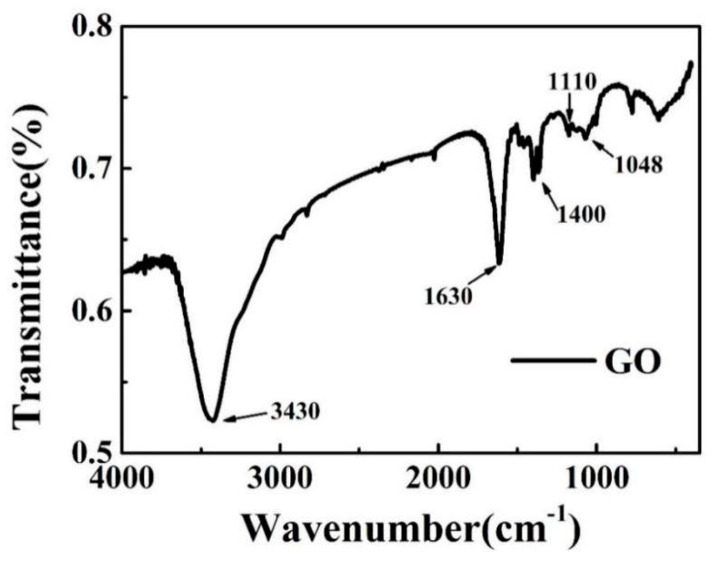
FTIR absorption spectra of the GO shown in Figure 1.

GO is a multifunctional material that contains numerous functional groups, such as epoxy (–O), hydroxyl (–OH), carboxyl (–COOH), and ether moieties. The FTIR spectrum of GO (Figure 2) contains peaks characteristic of O–H stretching vibrations (3430 cm^−1^), C–OH bending vibrations (1630 cm^−1^), saturated C–H bending vibrations (1400 cm^−1^), and asymmetric C–O–C stretching vibrations (1110 cm^−1^), showing it is rich in oxygen-containing functional groups.

### 3.2. Optimal Conditions for GO-Modified Sensor

**Figure 3:**
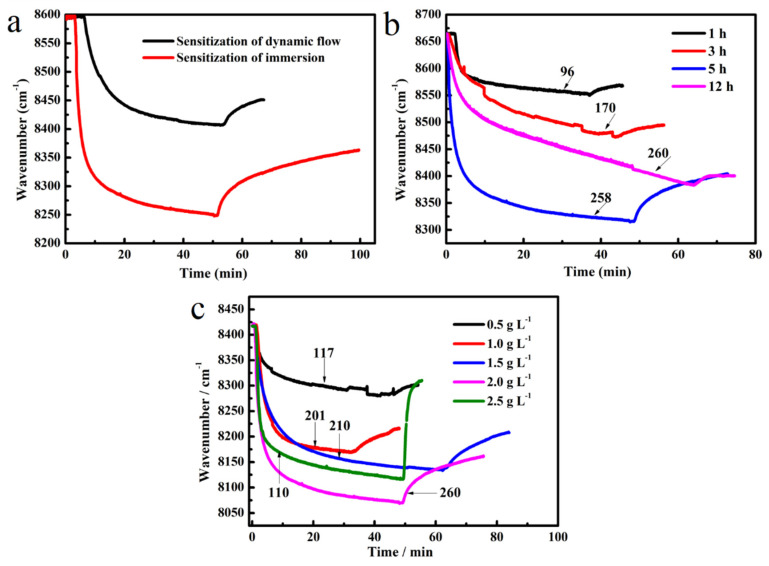
Exploration of optimal conditions for fabricating a GO-sensitized SPR biosensor. (**a**) SPR signal response for different GO modification methods, c(GO) = 2 mg/mL; (**b**) SPR signal response for various immersion time of gold flakes in GO aqueous solution (2 mg/mL); (**c**) SPR signal response to different concentrations of GO aqueous solution (0.5 to 2.5 mg/mL) after gold flakes immersed in GO aqueous solution for 5 h.

In this experiment, papain (500 mg/mL) was detected to explore the optimum conditions for SPR sensitization. By varying the modification modes, dynamic flow (flowing GO solution through the surface of the gold flake), immersion (immersing the gold flake in GO solution), modification time, and GO concentration, it was concluded that optimal sensitivity enhancement is obtained by immersion of the gold flakes (Figure 3a) for 5 h (Figure 3b) in 2.0 g/L GO (Figure 3c).

### 3.3. SEM Characterization of GO−Modified Sensor

**Figure 4:**
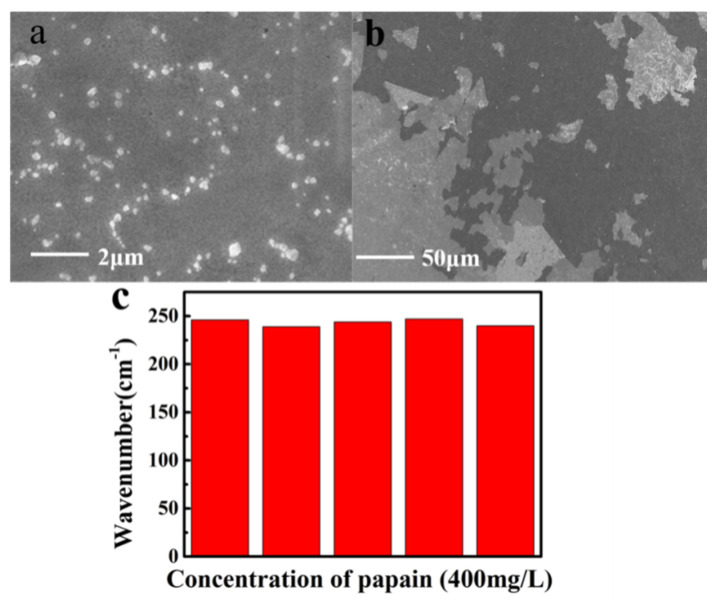
Characterization of GO−modified sensor (immersion for 5 h in 2.0 g/L^−1^ GO). (**a**,**b**) SEM images. (**c**) Reproducibility of the FT−SPR frequency change using a fixed papain concentration and GO-modified sensor under optimal conditions.

SEM images of the optimally modified GO−modified sensor (Figure 4a,b) show that the surface of the gold flake was successfully modified with GO. To determine whether the modification was reproducible, the preparation of the GO−modified sensor was repeated under the optimal conditions and used to detect a fixed concentration of papain. Figure 4c shows that there was no marked change in Fourier-transform SPR (FT-SPR) frequency, which indicated that the surface content of GO was reproducible from one preparation to the next.

### 3.4. GO-Sensitized SPR Detection of Papain

**Figure 5:**
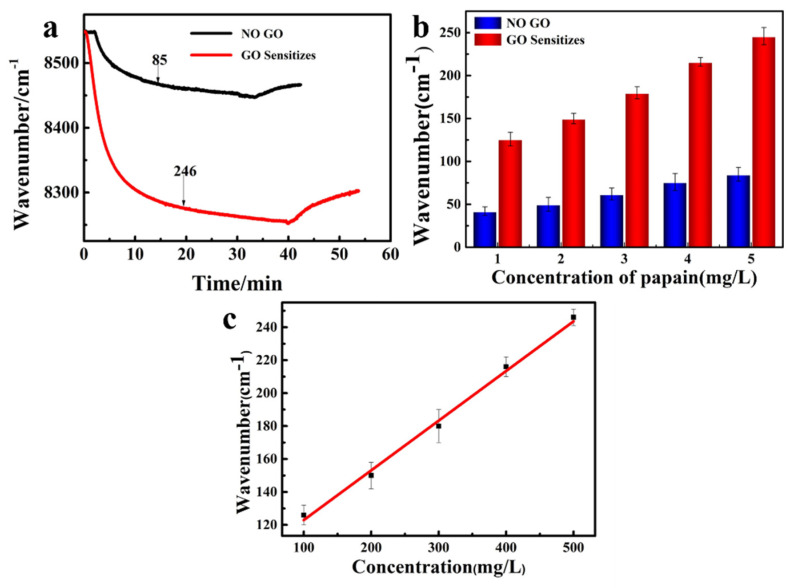
(**a**) Time dependence of FT−SPR signal in response to 500 mg/L papain, detected using sensors with and without GO modification. (**b**) Dependence of FT−SPR frequency change on papain concentration, measured using sensors with and without GO modification. (**c**) The linear relationship between papain concentration and FT−SPR frequency change of the GO-modified sensor (*R*^2^ = 0.994).

In the early work, as a mercapto protease enzyme, the papain has been reported to investigate the sensitization effect of different size noble metal nanoclusters [20]. Hence, GO-modified sensors under optimal conditions were used to detect different papain concentrations. Figure 5a compares the FT−SPR signal in response to 500 mg/L papain, using sensors with and without GO modification. The FT−SPR frequency change in response to 100–500 mg/L papain was also measured using sensors with and without GO modification. The SPR sensitivity was enhanced approximately 2.9-fold by the GO-modified sensor (Figure 5b), and a strong linear relationship (R^2^ = 0.994) between papain concentration and the FT−SPR frequency change was obtained (Figure 5c). The sensitizing effect of GO on SPR is that GO is modified to gold flake, which provides more binding sites and improves the sensitivity of SPR to substance detection. Considering that the surface of the bare gold of the chip has more binding sites than the GO−modified chip, we hypothesize that it is mainly due to the surface charge or the sensitizing effect of GO.

### 3.5. Application of the GO-Modified SPR Biosensor to PRRSV Detection

**Figure 6:**
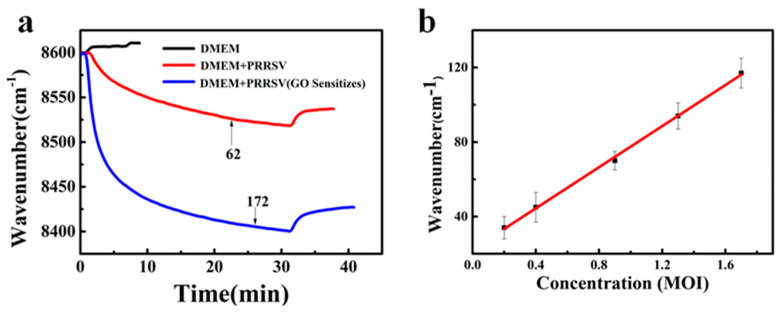
Specificity of porcine reproductive and respiratory syndrome virus (PRRSV) detection. (**a**) Time dependence of FT−SPR signal in response to PRRSV (2.6 MOI). (**b**) The linear relationship between the PRRSV concentration and the FT-SPR frequency change of the GO−modified sensor (*R*^2^ = 0.998). DMEM—Dulbecco’s modified Eagle medium; MOI—multiplicity of infection.

SPR biosensors with and without GO modification were cross-linked with CY functional peptides targeting PRRSV, and the FT−SPR signals in response to PRRSV were compared. As shown in Figure 6a, the addition of a PRRSV− free medium did not cause a signal change, indicating there were no matrix effects. Additionally, in the presence of PRRSV, the change in FT−SPR frequency of the GO-modified sensor was 2.8-fold larger than that of the unmodified sensor, showing that PRRSV and CY peptides can interact. As shown in Figure 6b, there is an obvious relationship between the signal intensity of the SPR sensor with the concentration of the PRRSV virus (*R*^2^ = 0.998) and the virus in the range of 0.4–1.7 MOI could be quantitatively detected.

### 3.6. Specificity of PRRSV Detection

**Figure 7:**
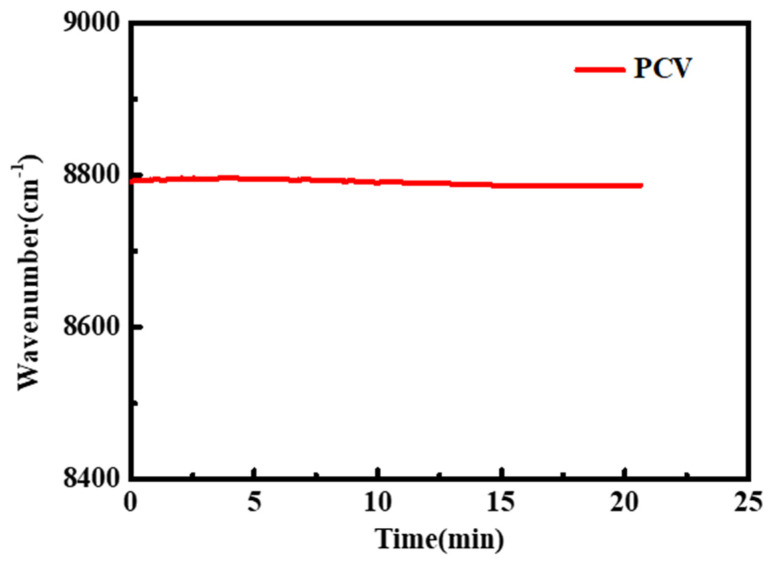
Time dependence of the FT−SPR signal after addition (arrow) of 2 MOI porcine circovirus (PCV).

To demonstrate that PRRSV specifically binds to CY peptides, porcine circovirus (2 MOI) was applied to the SPR biosensor under the same conditions used for PRRSV detection. As shown in Figure 7, the FT−SPR signal was essentially unchanged in response to porcine circovirus (PCV), indicating it had no binding interaction with the CY peptide.

## 4. Conclusions

In summary, a GO−modified SPR sensor was developed for fast, real-time detection of PRRSV. The GO−modified SPR chip has the advantages of simple operation, good plasmonic properties, real−time monitoring, and high sensitivity compared with traditional methods. By exploring different modification methods, optimal enhancement of the SPR sensitivity was obtained by immersion of gold flake in 2.0 g/L GO for 5 h. The GO−modified sensor under these conditions was confirmed using SEM and shown to enhance papain detection sensitivity 2.86−fold. The results of the TCID50 experiment were 10^−5.21^/0.1 mL. Compared with the traditional virus detection method, the prepared SPR sensor could realize the nondestructive and real-time online detection of viruses.

## Data Availability

Data is contained within the review article.
